# Differences between completely physically inactive and low active older men and their response to an exercise intervention: the Veterans LIFE study

**DOI:** 10.12715/har.2015.4.36

**Published:** 2015-09-07

**Authors:** Matthew J. Peterson, Carl F. Pieper, Richard Sloane, Gail M. Crowley, Patricia A. Cowper, Eleanor S. McConnell, Hayden B. Bosworth, Carola C. Ekelund, Megan P. Pearson, Katherine S. Hall, Miriam C. Morey

**Affiliations:** 1 Geriatric Research, Education and Clinical Center, Department of Veterans Affairs Medical Center, Durham, NC, USA; 2 Aging Center & Pepper OAIC, Duke University Medical Center; Durham, NC, USA; 3 Department of Medicine, Duke University Medical Center, Durham, NC, USA; 4 Department of Biometry and Bioinformatics, Duke University Medical Center, Durham, NC, USA; 5 School of Nursing, Duke University, Durham, NC, USA; 6 HSR&D, Department of Veterans Affairs Medical Center, Durham, NC, USA; 7 Department of Psychiatry and Behavioral Sciences, Duke University Medical Center, Durham, NC, USA

## Abstract

**Background:**

Physical activity interventions typically do not report behavioral changes in activity sub-groups. The aim of this study was to compare baseline differences and changes in physical activity between truly physically inactive men and low active men enrolled in a twelve-month, home-based physical activity intervention.

**Methods:**

Veterans with a mean age of 77.6 years were randomized to either a physical activity intervention or usual care. Measures included self-reported physical activity, physical function, and physical performance.

**Results:**

At baseline, the physically inactive group reported more symptoms and poorer functioning than the low active group. At 12 months, physically inactive men randomized to the intervention group increased their physical activity to an average of 73.3 minutes per week. Physically inactive individuals randomized to the control group were eight times more likely to remain inactive compared to the low active group.

**Conclusions:**

Completely physically inactive older men can markedly increase physical activity levels with a long-term intervention. Without such intervention, the likelihood of this group remaining inactive is eightfold.

## Introduction

Despite the numerous known health benefits of regular physical activity, chronic physical inactivity in the US remains a major public health problem [1, 2]. Poor fitness significantly increases mortality risk [3], and in a recent report it was estimated that 10% of all deaths worldwide can attributed to physical inactivity [4]. Further, research has shown that the greatest public health impact of physical activity promotion occurs when individuals with the lowest baseline physical activity status achieve a given increase in physical activity [5].

Factors associated with chronic physical inactivity are generally well understood. In a study of over 2,000 US adults, Martin and colleagues [6] reported that lesser perceived importance, being female, lower socioeconomic status, and older age predicted not meeting physical activity guidelines. While these factors remain important predictors of physical inactivity in old age, health factors such as diseases, their symptoms, and their impact on functioning are increasingly important in older adults’ compliance to regular physical activity [7, 8].

What have not been well described to date are the factors associated with being completely physically inactive in old age. Research reporting physical activity interventions have typically reported changes in physical activity as a group with no distinctions made between potential sub-group differences. The novel sub-group analyses presented in this paper will aid in our understanding of potential differences between subgroups of completely inactive versus low active older adults, and how exercise counseling affects these groups. Therefore, the purpose of this study was to compare baseline differences and responses to home-based exercise counseling between truly physically inactive men (i.e. 0 minutes of reported physical activity) and low active men (i.e. those with greater than 0 minutes of physical activity but who are not regularly active) enrolled in a 12-month, home-based exercise intervention.

## Methods

### Study design

The present study utilizes data from a completed clinical trial called the Veterans Learning to Improve Fitness and Function in Elders (Veterans LIFE) study. A complete description of the Veterans LIFE study has been previously published [9, 10]. In brief, the study was a randomized controlled trial comparing a one-year multi-component physical activity program to usual care. The study was reviewed and approved annually by the Durham Veterans Affairs (VA) institutional review board, and written consent was obtained from all patients prior to participation in the study.

### Participants

Enrollment in the Veterans LIFE study followed a detailed screening process. Initially, medical records were reviewed from all patients aged 70 and over who received primary medical care at the Durham VA Medical Center. Patients were excluded if they had a terminal diagnosis, unstable angina, history of ventricular tachycardia, chronic obstructive pulmonary disease requiring two hospitalizations within the previous 12 months, uncontrolled hypertension, stroke with moderate to severe aphasia, debilitating chronic pain, substance abuse, mental or behavioral disorder, dementia, or severe hearing or visual loss. The criteria were selected to maximize the safety of participants with multiple conditions undergoing an unsupervised home-based exercise program. A total of 3,995 medical records were examined, and recruitment packages were sent to 1,917 patients after receiving provider approval. Of these, 1,567 potentially eligible patients were contacted by telephone for recruitment into the study. During recruitment calls patients were further screened for eligibility by asking if they could walk 30 feet without human assistance, and whether they engaged in regular physical activity. Finally, 551 people came to the VA for an enrollment visit, and 400 consented to participate in the study and were randomized to a study group. The baseline visit included written informed consent, baseline assessments, and computer generated, block randomization.

The present study utilized baseline data from 392 of the 400 people originally enrolled in the Veterans LIFE study. Two female veterans enrolled in the original study were not included in this analysis; neither were six men with incomplete baseline measures.

### Definition of physically inactive and low active groups

For this analysis, groups were dichotomized in a manner to distinguish between individuals reporting absolutely no physical activity, and all others, given that - by study design - all eligible study participants were not meeting national guidelines for physical activity and would thus be classified as ‘low active’. Baseline reported weekly minutes spent in endurance and strengthening activities were assessed via the Community Healthy Activities Model Program for Seniors questionnaire (CHAMPS; see below). Physically inactive individuals reported no leisure time or exercise-specific physical activity (0 minutes). The low active group reported any minutes of leisure time or exercise-specific physical activity greater than 0 minutes, and no regular physical activity.

### Measures

The measures have been previously described in detail [9], and will be summarized below. All measures were collected at baseline and at three, six, and 12-month visits by a trained assistant blinded to randomization.

#### Physical activity

Physical activity information was collected using the CHAMPS activities questionnaire for older adults [11]. The CHAMPS questionnaire collects both frequency per week of all physical activities, and calories per week expended in all physical activities. The CHAMPS questionnaire has good construct validity, reliability, and is sensitive to change. We altered the questionnaire so that all activities were collected using minutes of reported activities as a continuous variable, rather than the original categorical variables described in CHAMPS, and were thus better able to capture small changes in minutes of physical activity.

#### Physical performance

Usual and rapid gait speed was measured on an eight-foot course with an electronic timing system (Speedtrap II). Other tests included the remaining two items of the Short Physical Performance Battery (SPPB: standing balance and timed chair stands) [12], and the Health, Aging and Body Composition-derived 400-meter long corridor walk test [13].

#### Self-reported symptoms, function and disability

A range of health symptoms prevalent in the aged were ascertained and summed to create a count of reported symptoms. Physical function, pain, and vitality were examined using the Medical Outcomes Study SF-36 questionnaire [14], and the Late Life Function and Disability Instrument (LL-FDI). The LL-FDI was used specifically to measure functional limitations and disability, and is reported to be sensitive to change [15].

#### Intervention

The intervention consisted of five distinct components. First, a health counselor met in person with each individual to construct a detailed physical activity plan with realistic short-term goals and a uniform long-term goal of accumulating 150 minutes/week in endurance activities and 45 minutes/week in strengthening activities. Second, the health counselor made regular telephone calls to each individual to reinforce the physical activity plan and to discuss health events, barriers, and set new short-term goals. Calls occurred every other week for the first six weeks and monthly thereafter. Third, each individual’s physician endorsed the prescribed physical activity plan in a primary care clinic visit. Fourth, pre-recorded automated telephone calls from the physician encouraging continued physical activity were delivered monthly to each individual. Lastly, materials were mailed quarterly providing personalized feedback regarding progress toward long-term goals.

#### Usual care

Individuals assigned to usual care received the standard care given to primary care patients in the primary or geriatrics clinics. Physical activity promotion varies widely between providers; some providers report assessing and counseling for physical activity at each clinic visit, while others do little or no physical activity counseling. VA mandates a yearly counsel recommending 30 minutes of physical activity on five days of the week.

### Statistical analysis

Baseline characteristics of the sedentary and low active groups were summarized as mean ± SD or frequencies. Characteristics between groups were compared using *t*-tests for continuous variables and chi-square tests for categorical variables. Dependent *t*-tests were used to test for significant changes in measures from baseline to 12 months within the groups. Logistic regression models estimated adjusted odds ratios for physically inactive participants reporting continued inactivity at study conclusion in the intervention and usual care groups.

## Results

### Baseline characteristics

At baseline there were significant differences between the physically inactive (*n*=159) and low active groups (*n*=233) in most measures (Table 1). Of note was the average weekly minutes spent in physical activity in the low active group at approximately 124 minutes/week, compared to no minutes in the physically inactive group (by definition). The large standard deviation observed in the low active group (±119 minutes/week) was due to high levels of reported time spent performing yard-work, gardening, or other non-exercise activities at the baseline interview, despite reporting not being regularly physically active during study inclusion screening. Physically inactive individuals had poorer mobility, physical performance and endurance, as measured by usual and rapid gait speed, SPPB score, and 400-meter walk time, respectively. Additionally the physically inactive group’s self-reported quality of life, functioning, disability and exercise efficacy were all worse than the low active group.

### Change in physical activity

In physically inactive individuals randomized to intervention, the weekly physical activity levels went from 0 at baseline to 73±88 minutes/week at 12 months (*p*<0.0001; Table 2). The mean 12-month change in weekly physical activity in physically inactive individuals randomized to the usual care group was also significant (19±50 minutes/week; *p*<0.001); and of sufficient magnitude to go from being below national guidelines for aerobic exercise to meeting guidelines. Low active individuals randomized to either the intervention or usual care groups had no significant changes in weekly physical activity at 12 months. After adjusting for age, education, race and number of self-reported symptoms at baseline, physically inactive individuals in the usual care group had significantly increased odds (OR=7.8; 95% CI: 3.4–17.9) of reporting sustained inactivity (0 minutes of weekly physical activity) at 12 months, compared to inactive individuals receiving the intervention (Table 3).

## Discussion

This study highlights three important findings: 1) the stark differences between individuals who are completely physically inactive and comparably aged men who engage in low amounts of physical activity; 2) tailored physical activity counseling is effective; and 3) individuals who are completely inactive most likely require a focused tailored physical activity intervention to initiate behavior change. We found that, at baseline, older men who were completely physically inactive had a health profile that placed them at greater risk for adverse health outcomes compared to the low active men in almost every baseline measure. For example, the physically inactive group’s usual gait speed was approximately 1.0 meter/second. This gait speed is considered a pre-clinical threshold for increased one-year risk of loss of function, worsening health status, and hospitalization [16]. The physically inactive group’s SPPB score of 9.0±2.3 is below the score (≤9.5) previously shown to be associated with increased likelihood of one-year hospitalization rates compared to those performing better on the test battery [16]. Additionally, the physically inactive group’s mean SF-36 scores in terms of physical function, pain, and vitality sub-scales are well below the 50^th^ percentile for men over 65 years old [17]. Knowledge of these characteristics and risks can aid health professionals in educating physically inactive older men on palpable adverse health outcomes associated with their lifestyle. Further, there is a growing body of literature indicating that emphasizing the negative impact of sedentary behavior may aid in behavior change [2].

An important finding from this study is that physically inactive older men respond favorably to a tailored, focused exercise intervention by significantly increasing their reported weekly physical activity levels. In this group there was an average improvement in weekly physical activity from 0 minutes/week to approximately 73 minutes/week with the intervention. While this weekly dose of physical activity does not meet minimal recommendations, there is emerging evidence that, compared to being physically inactive, as little as one hour of weekly physical activity confers a mortality benefit. Leitzmann et al. [18] described that older adults (mean age=62 years) who reported up to 1 hour/week in moderate intensity activities were at significantly lower risk for cardiovascular and cancer-related diseases, and all-cause mortalities compared to their physically inactive counterparts. In addition, new evidence suggests that older adults regularly engaging in non-exercise activities (e.g. cutting the lawn or performing home repairs) have reduced risk of cardiovascular events and mortality compared to those never engaging in these types of non-exercise activities. This reduction in risk was maintained after controlling for the effect of exercise-specific activities [19]. This reinforces the public health take-home message that there will be health benefits for older adults who are regularly active in any way that is enjoyable or meaningful to them.

We also examined if the probability of remaining completely physically inactive differed between intervention and usual care groups. Compared to physically inactive individuals receiving the intervention, those receiving usual care were almost eight times more likely to remain completely physically inactive at 12 months (adjusted OR=7.8; 95% CI: 3.4–17.9). This information suggests that despite the recent public health efforts encouraging physical activity and its many benefits, physically inactive older men are not likely to initiate a regular program of physical activity without a focused intervention. Data for North Carolina indicate a trend for increased prevalence of physical inactivity in older adults (30% in 2005 to 35% in 2009) [20]. New and innovative behavioral approaches are needed with this vulnerable segment of population.

Several components of the present study have great potential for future interventions and hospital-based programs aimed at physically inactive older adults. First, the tailored, in-person physical activity counseling session allowed the participant to take ‘ownership’ of his physical activity plan by communicating exercise preferences, self-scheduling the day, time, and place of exercise (as opposed to rigid facility-based programs), and by communicating concerns, fears and doubts with undertaking exercise. The health counselor facilitated the participant in navigating around barriers by educating him in the specific evidence regarding the benefits of physical activity linked to health concerns, and by setting attainable short-term goals (e.g. starting with five minute walks twice daily). The physical activity counseling materials are available at: www1.va.gov/resdev/resources/pubs/LIFE-modules.cfm.

Another important component of the intervention was the follow-up counseling calls. These calls facilitated progression of the physical activity program, and addressed inevitable roadblocks and barriers. Previous studies have employed telephone-based physical activity counseling with modest success [21, 22]. The telephone counseling piece in our study was highly motivational to the participants, as 90% of the intervention participants reported that the counseling calls motivated them to exercise ‘quite a bit’ or ‘very much.’

Lastly, physician involvement in the intervention was critical. The important role that physicians have in physical activity behavior change is well documented, yet physicians’ lack of time and expertise in prescribing exercise has resulted in widely variable practices in physician counseling [23, 24]. In the present study, physicians’ endorsement of the physical activity plan during the clinic visit was minimally time intensive (less than three minutes), largely due to the specifics of the plan already implemented by a trained exercise professional. The pre-recorded, automated physician telephone calls encouraging continued physical activity throughout the 12 months were viewed positively by both the physicians and participants. Overall the cost of physician time for each intervention participant was less than 12 US dollars.

There are limitations to this study. First, whether being physically inactive preceded or was a result of poor health is not known in this sample. Second, the implementation of the intervention at a VA hospital limits its generalizability, as veterans who receive health care at VA hospitals are generally in poorer health than the non-VA population [25]. Third, physical activity was assessed by self-report only. More objective measures, such as accelerometers, in lieu of self-report or as a validation tool would have been optimal.

## Conclusions

This study offers encouraging data to suggest that positive behavior change in physically inactive elderly men is possible. Despite poorer health, functioning, and exercise efficacy and motivation, elderly men who are truly physically inactive have the potential to dramatically increase their physical activity levels with a focused exercise counseling intervention. Conversely, without such intervention, the likelihood of this group remaining completely physically inactive is eightfold.

## Figures and Tables

**Table 1:** Baseline characteristics of the physically inactive and low active Veterans LIFE study participants

Measure	Physically Inactive (*n*=159)	Low Active (*n*=233)	*p*-value
Age	77.5 ± 5.1	77.5 ± 4.8	0.98
BMI (kg/m^2^)	29.5 ± 4.8	28.7 ± 4.5	0.11
Minutes/week in physical activity	0	123.8 ± 118.6	<0.0001
No. of symptoms	7.1 ± 4.6	6.2 ± 3.9	0.04
Physical performance			
Usual gait speed (m/s)	1.01 ± 0.25	1.08 ± 0.26	0.01
Rapid gait speed (m/s)	1.51 ± 0.42	1.63 ± 0.44	0.01
SPPB score	9.0 ± 2.3	9.6 ± 2.0	0.004
400 m. walk time (sec)	454.8 ± 10.7	406.8 ± 177.1	0.02
Self-report function and disability			
SF-36 physical function	61.1 ± 23.9	69.2 ± 22.1	0.006
SF-36 pain	64.9 ± 25.7	70.8 ± 24.9	0.02
SF-36 vitality	53.4 ± 20.9	60.4 ± 20.2	0.001
LL-FDI functional limitations	59.3 ± 11.2	61.7 ± 9.9	0.03
LL-FDI disability limitations	58.6 ± 5.6	60.9 ± 5.1	<0.0001

**Table 2:** Twelve-month changes in weekly physical activity in physically inactive and low active groups

	Baseline	12 months	*p*-value
Intervention			
Physically inactive (*n*=49)	0	73.3 ± 87.8	<0.0001
Low active (*n*=123) Control	140.9 ± 134.9	149.1 ± 155.8	0.60
Physically inactive (*n*=79)	0	19.1 ± 50.4	0.001
Low active (*n*=94)	104.5 ± 94.1	107.6 ± 133.5	0.83

**Table 3:** Odds of remaining physically inactive at 12-month follow-up

	Adjusted odds ratio*	95% confidence interval
Study group		
Intervention	1.00	referent
Control (*n*=79)	7.76	3.36–17.93
Age	0.99	0.91–1.08
Education		
Higher than high school	1.00	referent
High school	1.75	0.65–4.73
Less than high school	2.83	0.94–8.51
Race		
Caucasian	1.00	referent
Other	1.04	0.37–2.96
No. of symptoms at baseline	0.98	0.89–1.07

* All odds ratios adjusted for other variables shown in table
